# A Molecular Dynamics Study of a Photodynamic Sensitizer for Cancer Cells: Inclusion Complexes of γ-Cyclodextrins with C_70_

**DOI:** 10.3390/ijms20194831

**Published:** 2019-09-28

**Authors:** Giuseppina Raffaini, Fabio Ganazzoli

**Affiliations:** 1 Department of Chemistry, Materials, and Chemical Engineering “Giulio Natta”, Politecnico di Milano, Piazza L. Da Vinci 32, 20131 Milano, Italy; fabio.ganazzoli@polimi.it; 2 INSTM, National Consortium of Materials Science and Technology, Local Unit Politecnico di Milano, 20131 Milano, Italy

**Keywords:** fullerenes, C_70_, gamma-cyclodextrin, inclusion complexes, photosensitizer, molecular dynamics simulations, solubilization, photodynamic activity, cancer therapy, amphiphilic complexes

## Abstract

Photodynamic therapy is an emerging treatment of tumor diseases. The complexes with γ-cyclodextrins (γ-CD) and fullerenes or their derivatives can be used as photosensitizers by direct injection into cancer cells. Using molecular mechanics and molecular dynamics methods, the stability and the geometry of the 2:1 complexes [(γ-CD)_2_/C_70_] are investigated analyzing the differences with the analogous C_60_ complexes, studied in a previous theoretical work and experimentally found to be much less efficient in cancer therapy. The inclusion complex of γ-CD and C_70_ has a 2:1 stoichiometry, the same as C_60_, but is significantly less stable and displays an unlike arrangement. In vacuo, mimicking an apolar solvent, the complex is compact, whereas in water the two γ-CDs encapsulate C_70_ forming a relatively stable complex by interacting through their primary rims, however exposing part of C_70_ to the solvent. Other higher-energy complexes with the γ-CDs facing different rims can form in water, but in all cases part of the hydrophobic C_70_ surface remains exposed to water. The stability and arrangement of these peculiar amphiphilic inclusion complexes having non-covalent interactions in water can be an important key for cancer therapy to enhance both the solubilization and the fullerene insertion into liposomes or cell membranes.

## 1. Introduction

**Figure 1:**
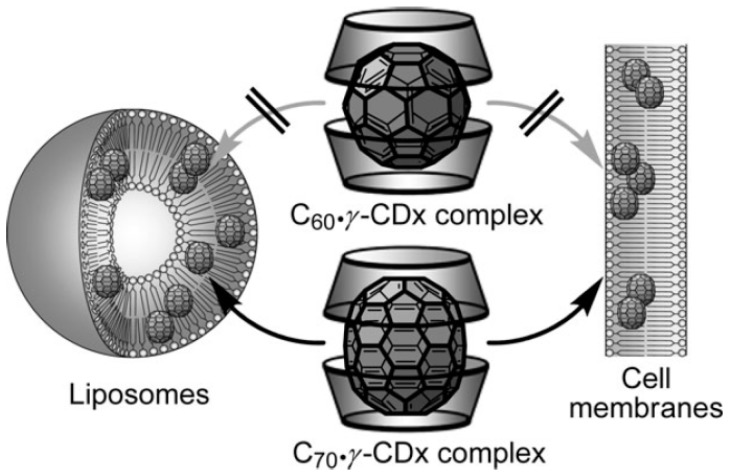
A sketch of the possible insertion of C_70_ (but not of C_60_) from the γ-CD complex into cell membranes or liposomes, as experimentally observed in [3]. Image reproduced with permission of Royal Society of Chemistry via the Copyright Clearance Center.

It was recently reported [1,2] that a photodynamic therapy can be used in the cancer therapy: the treatment is based on administration of a sensitizer and irradiation of target tissues by visible light after some delay necessary for the selective retention of the sensitizer. The singlet oxygen and other radical species formed directly in the tumor destroy it from inside. Ikeda et al. [3] proposed this picture, reported in Figure 1, in a schematic illustration of fullerenes (acting as sensitizers), encapsulated in a hydrophobic γ-cyclodextrin (γ-CD) cavity, displaying an exchange reaction to the liposomal and cell membranes [3] only in the case of C_70_, but not of C_60_.

Using molecular mechanics (MM) and molecular dynamics (MD) methods at the fully atomistic level, in this work at first the 1:1 complex [(γ-CD)/C_70_] and the dimer [(γ-CD)_2_/C_70_] are studied comparing these data with previous simulation results about these complexes with the fullerene C_60_ [4] in order to understand in primis the differences in geometry, stability, and hydration in water of the two complexes, and then the possible key factors for the crucial exchange reaction from the γ-CD complex to the liposomal and cell membranes for destroying a tumor or otherwise destroy the cancer cells.

Adopting the same simulation protocol proposed in the previous theoretical work [4] we studied the inclusion complexes both in vacuo, mimicking an apolar solvent, and in explicit water [5,6,7], using the consistent valence force field CVFF and the InsightII/Discovery packages [8]. The interaction between γ-CD and C_70_ is studied at first in a 1:1 stoichiometry, and then in the possible 2:1 stoichiometry. The formation of the more stable inclusion complexes is also studied in explicit water. In this case, we confirmed the stoichiometry of the inclusion complexes predicted in vacuo, but we found an opposite stability both for the initial 1:1 [(γ-CD)/C_70_] and for the final 2:1 [(γ-CD)_2_/C_70_] complexes, due to the formation of amphiphilic inclusion host-guest complexes, since part of the hydrophobic C_70_ surface remains accessible to water molecules. It is interesting to note that the amphiphilic nature of this 2:1 complex not only affects the complex stability through the interaction with the water molecules, but also the kinetics of possible release in contact with liposomes or cell membranes.

The study is carried out without any a priori assumption about the complex stoichiometry or geometry, and the most stable arrangement was obtained through the MD run considering the stepwise addition of two γ-CDs to C_70_ in different possible arrangements. The simulation protocol requires as a first step the study of the most stable geometry in vacuo considering the complex with a 1:1 host-guest stoichiometry, [(γ-CD)/C_70_], then considering the complex formation with a further γ-CD yielding a 2:1 host-guest stoichiometry, [(γ-CD)_2_/C_70_]. In the second step, the simulation protocol requires the same strategy for the complex formation in explicit water.

## 2. Results and Discussion

### 2.1. Complex Formation with a 1:1 Host-Guest Stoichiometry: [(γ-CD)/C_70_] In Vacuo

**Figure 2:**
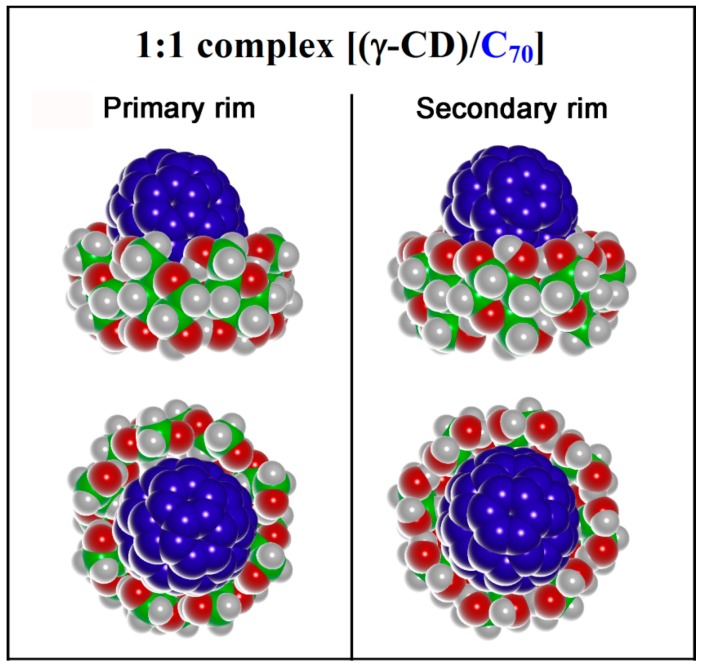
Side view and top view of the 1:1 complex [(γ-CD)/C_70_] formed by C_70_ with γ-CD in vacuo at the primary rim and at the secondary rim, in the most stable geometry found after MM and MD calculations. The γ-CDs are colored by atoms (C atoms are green, and O atoms are red, H atoms are in white, and the C_70_ atoms in blue).

At first two different interaction geometries between C_70_ and the primary or secondary rim of γ-CD, are considered, as reported in the supplementary in Figure S1. Using the same methodology proposed in previous work [3], after an initial energy minimization, an MD run in vacuo (i.e., an apolar solvent) lasting for 10 ns, and the minimization of numerous geometries saved during the MD run, the most favorable interaction geometries are reported in Figure 2 for the 1:1 host-guest stoichiometry [(γ-CD)/C_70_]. The same geometry was also obtained carrying out the simulations in explicit water.

In the most favorable complex, C_70_ interacts with the primary CD rim. In the isolated γ-CD, the primary rim is narrower than the secondary one, but it becomes considerably widened in the present case upon interaction with the fullerene. For instance, in the isolated γ-CD the diameter of the primary and secondary rims, defined as the average distance among the diametrically opposed O_6_ and O_3_ oxygens, respectively, amounted to 12.4 ± 1.7 Å (here the ± symbol refers to the standard deviation) and to 13.6 ± 0.8 Å in the order. On the other hand, upon complexation of C_70_ at the primary rim these two values became 13.2 ± 0.3 Å and 12.7 ± 1.4 Å (in the same order), so that the primary rim becomes the wider one with a narrower distribution of distances due to the geometrical constraint of the complexed fullerene. The most stable geometry exhibits a favorable interaction energy E_int_ = −258 kJ/mol, while the inclusion complex in which C_70_ interacts with the secondary γ-CD rim is less stable by 14.5 kJ/mol. It may be noted that the most stable arrangement displays an interaction energy that is more favorable by 17 kJ/mol compared to what found for the 1:1 complex between γ-CD with C_60_ [2]. Furthermore, it must be pointed out that C_70_ best interacts with the primary rim of γ-CD, as found for C_60_ in the most stable geometry for 1:1 complex.

**Figure 3:**
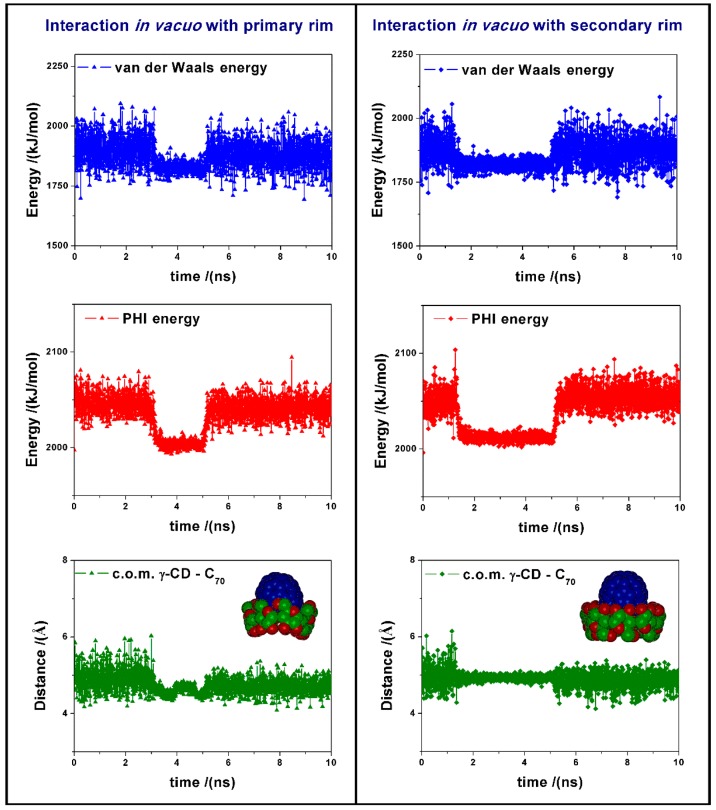
Van der Waals energy, PHI energy variation and distances between the c.o.m of *γ*-CD, and of C_70_ in the 1:1 complex [(γ-CD)/C_70_] calculated at 300 K as a function of time of MD runs carried out in vacuo for the initial interaction between C_70_ and the primary rim at left and for the initial interaction between C_70_ and the secondary rim at right.

During the MD run we observe changes in the van der Waals energy and in the torsional energy (PHI energy), as reported in Figure 3. These changes are due to the favorable interaction between the cyclodextrin and the fullerene, and to the variation in the dihedral angles of the γ-CD when the fullerene approaches the CD center. In vacuo the formation of the inclusion complexes is very fast, then we observe during the MD run some changes in the complex geometry and in parallel in the intermolecular van der Waals energy, together with final fluctuations around the average value from 5 to 10 ns. In this time range, the average potential energy of the 1:1 complex formed by interaction between C_70_ and the primary rim of γ-CD is equal to 7310.2 kJ/mol, while the complex with the secondary rim is less stable by 25.2 kJ/mol. Moreover, in the most stable complex the average distance between the center of mass (c.o.m.) of the fullerene and of the γ-CD, reported in Figure 3, is equal to 4.71 Å, whereas in the interaction with the secondary rim of γ-CD, this distance is slightly larger, being equal to 4.85 Å.

We can follow the inclusion process of the C_70_ fullerene interacting with the primary rim of the γ-CD and the inclusion process of C_70_ interacting with the secondary rim during the initial 5 ns of MD run in vacuo in the animation files reported in the links below Figure S1.

### 2.2. Complex Formation with a 2:1 Host-Guest Stoichiometry: [(γ-CD)_2_/C_70_] In Vacuo

**Figure 4:**
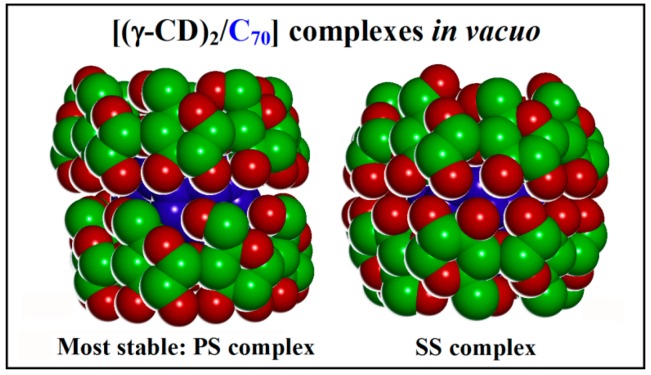
At left, the side view of the most stable inclusion complex in a 2:1 stoichiometry [(γ-CD)_2_/C_70_] formed in vacuo by the stable initial 1:1 [(γ-CD)/C_70_] complex facing it with another γ-CD through its secondary rim, so that eventually the two γ-CD’s interact through their primary and secondary rim, respectively (PS complex). At right, the side view of the less stable inclusion complex in a 2:1 stoichiometry [(γ-CD)_2_/C_70_] formed in vacuo facing the two secondary rim of the cyclodextrins (SS complex). The color code is the same of Figure 1. Here, the H atoms are omitted for clarity.

The possible complexes in a 2:1 stoichiometry [(γ-CD)_2_/C_70_] were modelled starting from four initial arrangements considering the two most stable geometries reported in Figure 2 and facing them with another γ-CD having either the primary or the secondary rim close to the exposed surface of C_70_ as reported in Figure S2 for clarity. Using the simulation protocol proposed in previous work [4,5,6,7], after MD runs lasting for 10 ns and optimization of numerous conformations assumed by the system, the most stable geometries are reported in Figure 4.

This most stable optimized geometry in vacuo for the PS complex (P refers to the first γ-CD in the most stable 1:1 stoichiometry and S to the second γ-CD) exhibits the lowest potential energy and also the most favorable interaction energy, equal to −593 kJ/mol. The SP complex, reported in Figure S3, was obtained by starting from the less stable 1:1 complex, so that P refers to the second CD: this complex has a very similar in conformation, being less stable than 10.0 kJ/mol, whereas the SS complex is less stable than 20.0 kJ/mol and finally the PP complex than 64.0 kJ/mol. During the MD run at 300 K from 5 ns to 10 ns, the difference between the average potential energy for the two PS and SS complexes reported in Figure 4 is even larger, amounting to 42.9 kJ/mol, thus confirming the significantly larger stability of the PS complex over the SS complex.

It should be pointed out that the C_60_ fullerene and γ-CD do also form the most stable complex with a 2:1 stoichiometry, but adopting an unlike geometry. In fact, C_60_ sits in a symmetrical cavity formed by two γ-CDs that interact through their wider secondary rims thanks to a large number of intermolecular H bonds, yielding a more favorable interaction energy of −652 kJ/mol. Conversely, C_70_ is found in a non-symmetrical cavity formed by the two γ-CDs that interact through the secondary rim of one CD (the upper one in Figure 4) and the primary rim of the other one (the lower one in Figure 4) thanks to some local distortions of the macrocycle. In turn, the interaction in the PS complex allows for a large number of H-bonds (7 or 8) among the OH groups on either CD so that the fullerene is well screened from the outer environment. It should be pointed out in this context that in the less stable SS complex the number of intermolecular H-bonds amounts to 16, so that all the secondary OH groups are involved in vacuo. On the other hand, optimization of these interactions involves different penalties to the two CDs, in particular a penalty in the torsional energy and in the non-bonded repulsive energy (poor contacts) that eventually destabilize the SS complex compared to the SP complex, as mentioned above. It is also of interest to note that the principal axis of the fullerene is slightly tilted with respect to the axis passing through the mean CD planes in the PS, SP, and PP complexes, whereas in the SS complex the two axes are perfectly aligned in this symmetric cavity.

We can follow the process of the complex γ-CD-C_70_ interacting with another γ-CD facing it with its primary or secondary rim, as reported in Figure S2, during the initial 5 ns of MD run in vacuo in the animation files reported in the links below Figure S2. From 5ns to 10 ns of the MD run, only fluctuations around the equilibrium positions are observed. The most stable complexes obtained in vacuo after minimization of fifty conformations periodically saved during MD run assumed by the system from 5 ns to 10 ns are reported in Figure S3.

### 2.3. Complex Formation of [(γ-CD)/C_70_] and then of the Dimer [(γ-CD)_2_/C_70_] in Water

As anticipated, the next step is to consider the geometry and the stability of the 1:1 and of the 2:1 complexes in explicit water. For this purpose, we studied the interaction between C_70_ and γ-CD adopting the same initial geometries as in vacuo (see Figure S1) but now with explicit water molecules in a box with periodic boundary conditions.

We can follow the inclusion process of the C_70_ fullerene interacting with the primary and with the secondary rim of the γ-CD during the MD run at 300 K in water lasting for 1 ns in the animation files (see links reported before Figure S4 in Supplementary Materials).

**Figure 5:**
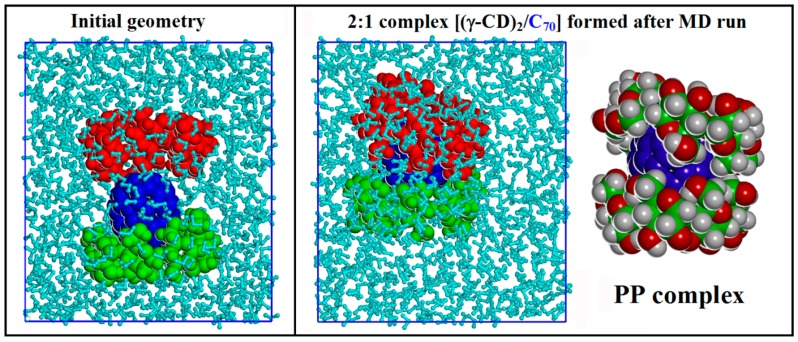
The complex γ-CD with C_70_ in a stoichiometry 1:1 in water facing the primary rim of the second γ-CD (at left), to study the formation of the complex in a 2:1 stoichiometry after 2 ns of MD run in explicit solvent (at right). The most stable geometry obtaind for the complex where the CD face their primary rims (PP complex) is also shown after removing the water molecules for clarity.

The inclusion of hydrophobic C_70_ in the hydrophobic cavity of γ-CD is very fast and the complex formed is stable during the MD run. The most stable geometry for the 1:1 complex [(γ-CD)/C_70_] corresponds to the favorable interaction between the fullerene and the secondary rim of γ-CD, unlike to what was found in vacuo. Starting from this 1:1 complex and facing it in water with the second γ-CD in the same arrangements reported in Figure S2 in SI, as well as starting from the less stable 1:1 complex with the interaction of the fullerene at the primary CD rim, we studied the formation of 2:1 host-guest stoichiometry inclusion complexes. After initial geometry optimizations using MM methods and then an MD run at 300 K lasting for 2 ns in explicit water, the final results show that the most stable host-guest stoichiometry in water corresponds again to the 2:1 complex. However, in water the most stable arrangement, reported at right in Figure 5, shows that C_70_ is hosted in the cavity formed by the two γ-CDs that interact through their primary rims without any intermolecular H bonds, as reported in Figure S4. In fact, a large number of H bonds are present, but they only involve the terminal OH groups and the water molecules of the first hydration shell of the complex.

It is important to note that the most stable inclusion complex with a 2:1 stoichiometry [(γ-CD)_2_/C_70_] in water is the PP complex shown in Figure 5, followed by the SS complex, which is less stable by 10.8 kJ/mol, and then by the SP complex, less stable by 18.6 kJ/mol. In water the stability of the three possible inclusion complexes is different from that obtained by the simulation in vacuo described before. The complexes formed in water are very peculiar and the arrangements of the two γ-CDs that include C_70_ is very different with respect to that achieved with C_60_. This difference in the inclusion complex geometry potentially has important consequences for the release of the fullerenes in amphiphilic liposomes or in cell membranes. In fact, in the case of C_70_ the two γ-CDs rims approach only in part at the equatorial plane of the complex and do not form intermolecular H-bonds due to presence of the prolate fullerene in the cavity (see Figure S4), unlike what was found for C_60_. As a result, part of the hydrophobic C_70_ surface is still exposed to the water molecules. All 2:1 host-guest complexes [(γ-CD)_2_/C_70_] formed in water are then amphiphilic inclusion complexes.

**Figure 6:**
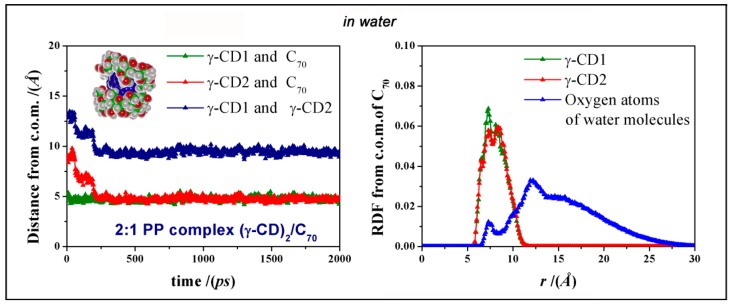
At left, the distance calculated during MD run in water between the c.o.m. of C_70_ and of the first γ-CD (the lower CD in the inset) in green, between the c.o.m. of C_70_ and of the second γ-CD (the upper CD) in red, and the distance between the c.o.m. of the two γ-CDs in blue for the PP complex. At right, the radial distribution function RDF calculated as a function of the distance r from the fullerene c.o.m of the atoms of two different γ-CDs of the PP [(γ-CD)_2_/C_70_] complex and of oxygen atoms of the water molecules.

**Figure 7:**
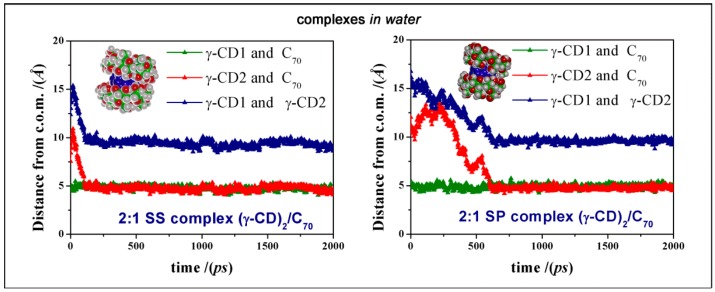
The same as in Figure 6 at left for the SS and SP [(γ-CD)_2_/C_70_] complexes.

We also add that the most favorable geometry PP complex is stable during the MD run, as shown by the graphic reported in Figure 6, showing that the second γ-CD approaches the initial complex formed between one γ-CD and C_70_ and both remain at the same distance from the C_70_ c.o.m. during the rest of MD simulation. A similar behavior was also found for the less stable SS and SP complexes, as reported in Figure 7. In Figure 6, at right, we also show the radial distribution function (RDF) of all the atoms of the two γ-CDs in the PP complex and of the oxygen atoms of water molecules measured as a function of the distance r from the c.o.m of C_70_, calculated during the MD run from 1 to 2 ns. The distribution of the two γ-CDs is very similar, while the water molecules have a weak maximum at 7.3 Å from the C_70_ c.o.m near the exposed surface of the fullerene. Moreover, the peak at 11.9 Å is the first hydration shell of the two cyclodextrins. Similar RDFs were found for the other amphiphilic complexes formed in water.

The stability of the most favorable geometry, where the two CD’s interact through their primary rims, is related to the favorable van der Waals interaction of the fullerene with the hydrophobic CD cavities but also by a number of the H-bonds among water molecules and the primary rims of two cyclodextrins, in addition to those formed within each γ-CD. For the PP [(γ-CD)_2_/C_70_] complex we note favorable H-bonds involving water molecules and the primary rims. The water molecules do form a bridge between the OH of the two CD’s when their distance is not too large. As a consequence, the most stable geometry is less constrained than what was observed in the case of C_60_, where the number of intermolecular H-bonds is maximized. Therefore, in the amphiphilic complex thanks to these non-covalent interactions with C_70_ the overall arrangement has the important possibility to open up in an appropriate environment. In this context, we additionally note that the other, less stable complexes show very few intermolecular H-bonds, involving spatially close OH groups of two cyclodextrins (see Figure S4). Finally, we point also out that these geometries are not static, because the average planes through the CDs change their tilt angle with respect to the main axis of the fullerene, so that the groups that come into contact through H-bonds change continuously moving around the two rims.

Moreover, we can observe that all the complexes formed in water, as reported in Figure S5a–c, have similar radii of gyration and solvent accessible surfaces. The PP and SS [(γ-CD)_2_/C_70_] complexes have similar dipole moment vectors, a bit inclined or roughly parallel to the equatorial plane separating the two CDs, while the less stable SP complex has a dipole moment vector roughly perpendicular to it with a larger moment. This difference in the dipole moment vector can be important when these amphiphilic complexes will be in contact with the polar surfaces of liposomes or cell membranes, because it affects the complex orientation, favoring the interaction with the exposed fullerene surface in the PP (and SS) complex.

We can follow the formation of the 2:1 complex when the 1:1 complex where C_70_ interacts with the secondary rim of the first γ-CD is approached by the secondary rim or by the primary of the second γ-CD (respectively, the left and at right of Figure 7 and in Figure S4), during the initial MD run in water in the animations files in Supplementary Materials (see the links reported below Figure S4 in the Supplementary Materials). The same information about the most stable PP [(γ-CD)_2_/C_70_] complex in water is also reported in the Supplementary Materials.

## 3. Materials and Methods 

The simulations were performed with the InsightII/Discover packages [8] using the consistent valence force field CVFF. For the 1:1 [(γ-CD)/C_70_] complexes the size of the cubic cell containing about nine hundred water molecules was 31 × 31 × 31 Å; for the 2:1 [(γ-CD)_2_/C_70_] complexes, the cell containing about eight hundred water molecules had cell parameters equal to 31 × 35 × 32 Å. The structure of C_70_, taken from the solid-state X-ray analysis, is available in the Materials Studio [8] simulation program, while the CD structure was obtained as described in [7] using the Builder module of InsightII/Discover.

All the energy minimizations were carried out up to an energy gradient lower than 4 × 10^−3^ kJ mol^−1^ Å^−1^ both for the initial simulations in vacuo and in the presence of the explicit water molecules. The MD runs were performed at a constant temperature (*T* = 300 K) controlled through the Berendsen thermostat and adopting a non-bonded cut off of 17 Å. Integration of the dynamical equations was carried out with the Verlet algorithm using a time step of 1 fs, and the instantaneous coordinates were periodically saved for further analysis. In vacuo the length of the MD runs was 10 ns and in water 1 ns for the 1:1 [(γ-CD)/C_70_] complex using the periodic boundary condition, and 2 ns for the 2:1 [(γ-CD)_2_/C_70_] complex. Within the MD runs, we monitored both the time changes of the total and potential energy together with its components, and the changes in the distance between the centers of mass (c.o.m) of the cyclodextrins and of the fullerene. In general, these quantities showed an initial decrease, possibly with a few separate kinetic stages, and then fluctuated around a constant value, indicating achievement of equilibrium, which required adopting different lengths of the dynamic trajectories in different environment. Using MM and MD methods and adopting the simulation protocol proposed by us [4,5,6,7] we can, thus, study both the kinetics of inclusion and the formation and stability of the complexes when an equilibrium state was achieved. In particular, in vacuo, in order to study the most stable system, fifty geometries saved periodically during the MD run from 5 ns and 10 ns were optimized. In water, at the end of MD run lasting 2 ns, when equilibrium was achieved, the final geometry was optimized. The equilibrium geometries sampled during an MD run, including the distances between selected sets of atoms, are best analyzed through the radial distribution function (or simply RDF) calculated from the trajectories of the simulations. The RDF gives the probability density of finding atoms *j* (for instance in Figure 6, the atoms of two γ-CDs or the oxygen atoms of the water molecules) at a distance r from atoms *i* (or from a specific point such as the c.o.m. of the C_70_ as reported for instance in Figure 6).

## 4. Conclusions

In this paper, we report a fully atomistic simulation study of the interaction between γ-CD and the C_70_ fullerene carried out with molecular mechanics (MM) and molecular dynamics (MD) methods. We find that in vacuo, mimicking an apolar solvent, the most stable 1:1 [(γ-CD)/C_70_] complex is similar in stability and in arrangement to what found for the analogous C_60_ complex, with a good interaction of the fullerene with the γ-CD primary rim. Conversely, in water the stability is different, since the most stable 1:1 [(γ-CD)/C_70_] complex involves non-covalent interactions between the γ-CD secondary rim and C_70_.

When the concentration of γ-CD increases, the 2:1 [(γ-CD)_2_/C_70_] complex is formed, and for this host-guest stoichiometry we found different arrangements having C_70_ well encapsulated in the hydrophobic cavity formed by the γ-CDs. However, the most stable 2:1 inclusion complexes display a mutual arrangement of the two γ-CDs strongly different with respect to the same complex with C_60_ previously investigated [4]. In fact, in vacuo the most stable 2:1 complex including C_70_ involves the interaction between the primary rim of one γ-CD and the secondary rim of the other one, while other CD arrangements are much less stable. In all cases, anyway, in vacuo all these complexes are quite compact and the fullerene is almost inaccessible to the outer environment. On the other hand, in water the most stable 2:1 inclusion complex with C_70_ involves the interaction among the two γ-CDs through their primary rims, which is a most unusual geometry in CD host-guest complexes. Other less stable geometries are possible, corresponding to the interaction between the two γ-CD secondary rims or between one secondary and one primary rim in order of increasing energy.

Therefore, the interaction of γ-CD and C_70_ in water leads to the formation of very interesting amphiphilic complexes thanks to non-covalent interactions with an unlike stability than in an apolar solvent both for the 1:1 and the 2:1 complexes. The most stable complex is the dimeric [(γ-CD)_2_/C_70_] with a 2:1 stoichiometry, as already found with C_60_ [4,9,10], but the two fullerenes involve strongly different arrangements of the two CDs. In fact, in the most stable dimeric complex the two γ-CDs interacting through the primary rims are well hydrated, but do not form any intermolecular hydrogen bond, and still expose to water part of the fullerene surface. On the other hand, in the smaller and more spherical C_60_ fullerene the most favorable arrangement involves the strong interaction of the two γ-CDs through their secondary rims, thus optimizing the intermolecular hydrogen bonds, leading to a very tight and stable complex with C_60_.

**Figure 8:**
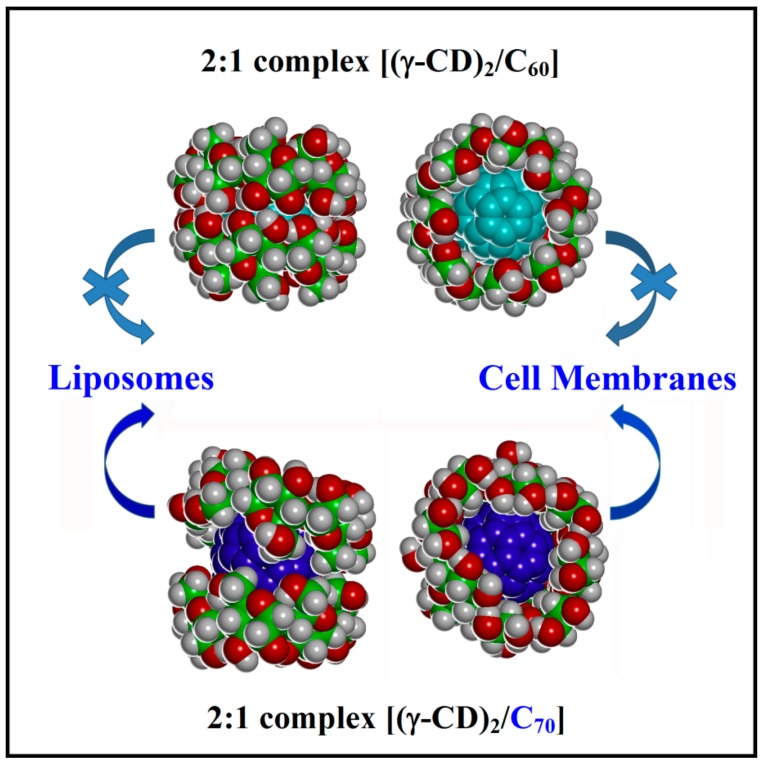
A sketch of the [(γ-CD)_2_/C_70_] complex structure as obtained in the present theoretical work and of the analogous [(γ-CD)_2_/C_60_] complex from [4] summarizing also the unlike experimental behavior [3]: the former complex exposes part of the hydrophobic fullerene surface than the latter one, and accordingly it is more prone to release the C_70_ fullerene when in contact with the liposomes or cell membranes, as also shown in the sketch of Figure 1 based on experimental data [3].

A most important result of the present simulations, however, is that the two γ-CDs, which are essential to solubilize fullerenes in water [11], do not completely shield the hydrophobic C_70_ from the outer environment, unlike what found for C_60_. Moreover, the inclusion complexes are not tight and particularly rigid: rather, the CDs are quite mobile around the main axis of C_70_, while their average planes undergo also a tilting motion about the same axis. This observation can be relevant to enhance the interaction and the release of C_70_ close to liposomes or cell membranes [12], unlike what happens with C_60_ as experimentally found in [3]. Figure 8 summarizes these results and replaces the sketch reported in Figure 1 by drawing the correct arrangements of the CDs in the two complexes as obtained in the present work.

Further simulations about the interaction of these amphiphilic complexes with a cell membrane model will be important in order to study the possible release of the hydrophobic fullerene solubilized in water thank to these amphiphilic systems. Finally, we also note that we considered only native CDs, while the use of covalently modified CDs is sometimes adopted, in order to achieve also the formation of supramolecular amphiphilic aggregates for the controlled drug delivery of hydrophobic drugs [13]. Such systems, their enhanced complex formation and their aggregation behavior will be considered in future work.

## Supplementary Materials

Supplementary materials can be found at https://www.mdpi.com/1422-0067/20/19/4831/s1. Figure S1: Side view of the initial non-optimized geometries in vacuo of C_70_ at left near the primary rim and at right near the secondary rim of γ-CD. The carbon atoms are in green, the oxygen atoms in red; the hydrogen atoms are omitted for clarity.; Figure S2: Side view of the initial four non optimized geometries in vacuo, starting the complex with the 1:1 stoichiometry [(γ-CD)/C_70_] in the optimized geometry at the primary (top) and at the secondary rim (bottom), and facing it with a second γ-CD with the primary (at left) and the secondary rim (at right), as indicated in the two panels.; Figure S3: Side view of the most stable geometries of 2:1 Host-Guest Stoichiometry [(γ-CD)_2_/C_70_] found after the MD runs in vacuo at 300 K and optimization of numerous conformations (50 conformations periodically saved during the MD run), starting from the initial complexes in the 1:1 stoichiometry (Figure S3), interacting with two different rims of the second γ-CD. Hydrogen atoms are omitted for clarity. See Figure S2 for the color codes; Figure S4: Stick side view of the final geometries obtained after MD runs lasting for 2 ns in water at 300 K and optimization of the conformation at equilibrium for the 2:1 complexes [(γ-CD)_2_/C_70_]. Hydrogen bonds are in white dotted lines. Water molecules and the simulation cells are omitted for clarity. The SS and SP complexes are reported using also the CPK representation. See Figure S2 for the color codes; Figure S5a. Information about PP [(γ-CD)_2_/C_70_] complex in water. In the box, the values of the radius of gyration, Rg, of the solvent accessible surface area and the dipole moment are reported. The figures show the dipole moment (on the top at left), the solvent accessible surface in the side view (on the top at right) and in the top views from the two secondary rims (below).; Figure S5b: Information about SS [(γ-CD)_2_/C_70_] complex in water. In the box, the values of the radius of gyration, Rg, of the solvent accessible surface area and the dipole moment are reported. The figures show the dipole moment (on the top at left), the solvent accessible surface in the side view (on the top at right) and in the top views from the two secondary rims (below); Figure S5c: Information about SP [(γ-CD)_2_/C_70_] complex in water. In the box, the values of the radius of gyration, Rg, of the solvent accessible surface area and the dipole moment are reported. The figures show the dipole moment (on the top at left), the solvent accessible surface in the side view (on the top at right) and in the top views from the two secondary rims (below).

## Abbreviations

MM	Molecular Mechanics
MD	Molecular Dynamics
γ-CD	γ-Cyclodextrin
PP	2:1 [(γ-CD)_2_/C_70_] complex formed by facing the Primary and the Primary rim of two γ-CDs
PS	2:1 [(γ-CD)_2_/C_70_] complex formed by facing the Primary and the Secondary rim of two γ-CDs
SS	2:1 [(γ-CD)_2_/C_70_] complex formed by facing the Secondary and the Secondary rim of two γ-CDs
c.o.m.	Center of mass
RDF	Radial distribution function
R_g_	Radius of gyration
