# Supplementary material for: A Molecular Dynamics Study of a Photodynamic Sensitizer for Cancer Cells: Inclusion Complexes of γ-Cyclodextrins with C70
Source: Int J Mol Sci. 2019 Sep 28;20(19):4831. doi: 10.3390/ijms20194831 (PMC6801912; doi:10.3390/ijms20194831)
Supplement: Supplementary file 1 [file ijms-20-04831-s001.zip › ijms-589888-supplementary.docx]

Supplementary for:

A Molecular Dynamics Study of a Photodynamic Sensitizer for Cancer Cells: Inclusion Complexes of γ-Cyclodextrins with C_70_

Giuseppina Raffaini ^1,2,^* and Fabio Ganazzoli ^1,2^

^1^ Department of Chemistry, Materials, and Chemical Engineering “Giulio Natta”, Politecnico di Milano, Piazza L. Da Vinci 32, 20131 Milano (Italy);

^2^ INSTM, National Consortium of Materials Science and Technology, Local Unit Politecnico di Milano, Milano, Italy.

***** Correspondence: giuseppina.raffaini@polimi.it ; Tel.: +39-0223993068.

Table of Contents

Complex Formation with a 1:1 Host-Guest Stoichiometry: [(γ-CD)/C_70_] *in vacuo*

**Figure S1**. Side view of the initial non-optimized geometries in vacuo of C_70_ at left near the primary rim and at right near the secondary rim of γ-CD. The carbon atoms are in green, the oxygen atoms in red; the hydrogen atoms are omitted for clarity.………………………………...……………………...…..page S3

We can follow the inclusion process of the C_70_ interacting with the primary rim of the γ-CD and the inclusion process of fullerene intercting with the secondary rim during the initial 5 ns of MD run in vacuo in the the animations:

[dyn_in vacuo_Gcd primary rim_C70_5ns.avi](file:///C:\Users\ganazzoli.CHKNET\Documents\Ciclodestrine\Fullereni_inclusi\PaperC70\dyn_in%20vacuo_Gcd%20primary%20rim_C70_5ns.avi)

[dyn_in vacuo_Gcd secondary rim_C70_5ns.avi](file:///C:\Users\ganazzoli.CHKNET\Documents\Ciclodestrine\Fullereni_inclusi\PaperC70\dyn_in%20vacuo_Gcd%20secondary%20rim_C70_5ns.avi), respectively.

Complex Formation with a 2:1 Host-Guest Stoichiometry: [(γ-CD)_2_/C_70_] *in vacuo*

**Figure S2**. Side view of the initial four non optimized geometries in vacuo, starting the complex with the 1:1 stoichiometry [(γ-CD)/C_70_**]** in the optimized geometry at the primary (top) and at the secondary rim (bottom), and facing it with a second γ-CD with the primary (at left) and the secondary rim (at right), as indicated in the two panels…................................................................................................................page S4

We can follow the process of the complex γ-CD - C_70_ interacting with another γ-CD facing it with the primary and with the secondary rim as reported in Figure S2, during the initial 5 ns of MD run in vacuo in the animation:

[DYN_dimer_gCDs_C70_PP_5ns.avi](file:///C:\Users\ganazzoli.CHKNET\Documents\Ciclodestrine\Fullereni_inclusi\PaperC70\DYN_dimer_gCDs_C70_PP_5ns.avi),

[DYN_dimer_gCDs_C70_PS_5ns.avi](file:///C:\Users\ganazzoli.CHKNET\Documents\Ciclodestrine\Fullereni_inclusi\PaperC70\DYN_dimer_gCDs_C70_PS_5ns.avi),

[DYN_dimer_gCDs_C70_SP_5ns.avi](file:///C:\Users\ganazzoli.CHKNET\Documents\Ciclodestrine\Fullereni_inclusi\PaperC70\DYN_dimer_gCDs_C70_SP_5ns.avi),

[DYN_dimer_gCDs_C70_SS_5ns.avi](file:///C:\Users\ganazzoli.CHKNET\Documents\Ciclodestrine\Fullereni_inclusi\PaperC70\DYN_dimer_gCDs_C70_SS_5ns.avi), respectively.

**Figure S3**. Side view of the most stable geometries of 2:1 Host-Guest Stoichiometry [(γ-CD)_2_/C_70_**]** found after the MD runs in vacuo at 300 K and optimization of numerous conformations (fifty conformations periodically saved during the MD run), starting from the initial complexes in the 1:1 stoichiometry (Figure S3), interacting with two different rims of the second γ-CD. Hydrogen atoms are omitted for clarity. See Figure S2 for the color codes…………....................................................….....page S5

Complex Formation with a 1:1 Host-Guest Stoichiometry: [(γ-CD)/C_70_] *in water*

We can follow the inclusion process of the C_70_ fullerene interacting with the primary and with the secondary rim of the γ-CD during the MD run in water lasting for 1 ns in the animation files

[dyn_in water_Gcd primary rim_C70.avi](file:///C:\Users\ganazzoli.CHKNET\Documents\Ciclodestrine\Fullereni_inclusi\PaperC70\dyn_in%20water_Gcd%20primary%20rim_C70.avi)

[dyn_in water_Gcd secondary rim_C70.avi](file:///C:\Users\ganazzoli.CHKNET\Documents\Ciclodestrine\Fullereni_inclusi\PaperC70\dyn_in%20water_Gcd%20secondary%20rim_C70.avi), respectively……………………………………...............page S6

Complex Formation with a 2:1 Host-Guest Stoichiometry: [(γ-CD)_2_/C_70_] *in water*

**Figure S4**. Stick side view of the final geometries obtained after MD runs lasting for 2 ns in water at 300 K and optimization of the conformation at equilibrium for the 2:1 complexes [(γ-CD)_2_/C_70_]. Hydrogen bonds are in white dotted lines. Water molecules and the simulation cells are omitted for clarity. Only for the SS and SP complexes are reported using also the CPK representation. See Figure S2 for the color codes….…………………………………..……………………….……………..........……..page S7

We can follow the formation of the 2:1 complex when the 1:1 complex, where C_70_ interacts with the secondary rim of the first γ-CD, is approached by the secondary or by the primary rim of the second γ-CD (respectively at left and at right of Figure 7 and in Figure S4), during the initial MD run in water in the animations files [DIM_SS_in water_500ps.avi](file:///C:\Users\ganazzoli.CHKNET\Documents\Ciclodestrine\Fullereni_inclusi\PaperC70\DIM_SS_in%20water_500ps.avi), [DIM_SP_in water_1ns.avi](file:///C:\Users\ganazzoli.CHKNET\Documents\Ciclodestrine\Fullereni_inclusi\PaperC70\DIM_SP_in%20water_1ns.avi), respectively. The same information about the most stable PP [(γ-CD)_2_/C_70_] complex in water as reported in Figure 6 is in the file [DIM_PP_in water_1ns.avi](file:///C:\Users\ganazzoli.CHKNET\Documents\Ciclodestrine\Fullereni_inclusi\PaperC70\DIM_PP_in%20water_1ns.avi).

**Figure S5a**. Information about PP [(γ-CD)_2_/C_70_] complex in water. In the box, the values of the radius of gyration, R_g_, of the solvent accessible surface area and the dipole moment are reported. The figures show the dipole moment (on the top at left), the solvent accessible surface in the side view (on the top at right) and in the top views from the two secondary rims (below).……...................................page S9

**Figure S5b**. Information about SS [(γ-CD)_2_/C_70_] complex in water. In the box, the values of the radius of gyration, R_g_, of the solvent accessible surface area and the dipole moment are reported. The figures show the dipole moment (on the top at left), the solvent accessible surface in the side view (on the top at right) and in the top views from the two secondary rims (below).........................….........…page S10

**Figure S5c**. Information about SP [(γ-CD)_2_/C_70_] complex in water. In the box, the values of the radius of gyration, R_g_, of the solvent accessible surface area and the dipole moment are reported. The figures show the dipole moment (on the top at left), the solvent accessible surface in the side view (on the top at right) and in the top views from the two secondary rims (below).......................... ...............page S11

Complex Formation with a 1:1 Host-Guest Stoichiometry: [(γ-CD)/C_70_] *in vacuo*


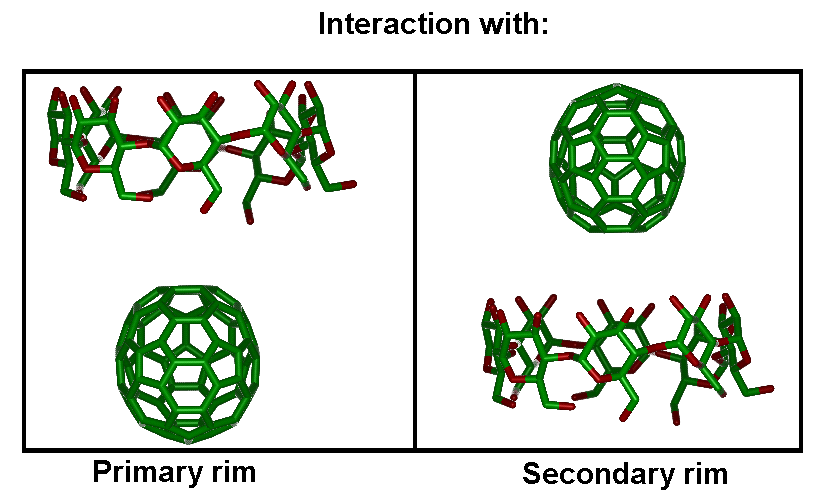


**Figure S1**. Side view of the initial non-optimized geometries in vacuo of C_70_ at left near the primary rim and at right near the secondary rim of γ-CD. The carbon atoms are in green, the oxygen atoms in red; the hydrogen atoms are omitted for clarity.

We can follow the inclusion process of the C_70_ interacting with the primary rim of the γ-CD and the inclusion process of fullerene intercting with the secondary rim during the initial 5 ns of MD run in vacuo in the animations:

[dyn_in vacuo_Gcd primary rim_C70_5ns.avi](file:///C:\Users\ganazzoli.CHKNET\Documents\Ciclodestrine\Fullereni_inclusi\PaperC70\dyn_in%20vacuo_Gcd%20primary%20rim_C70_5ns.avi)

[dyn_in vacuo_Gcd secondary rim_C70_5ns.avi](file:///C:\Users\ganazzoli.CHKNET\Documents\Ciclodestrine\Fullereni_inclusi\PaperC70\dyn_in%20vacuo_Gcd%20secondary%20rim_C70_5ns.avi), respectively.

Complex Formation with a 2:1 Host-Guest Stoichiometry: [(γ-CD)_2_/C_70_] *in vacuo*


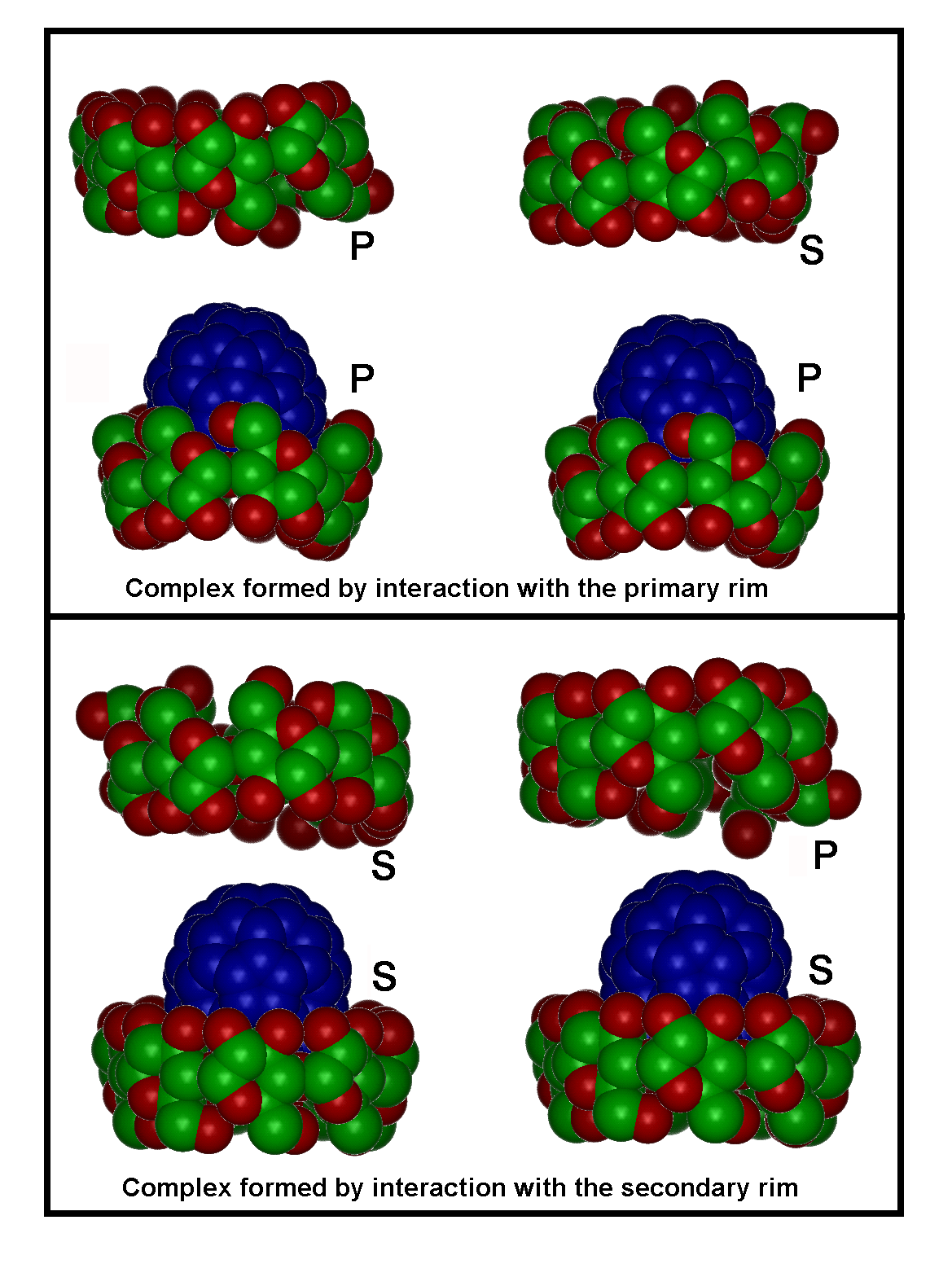


**Figure S2**. Side view of the initial four non optimized geometries in vacuo, starting the complex with the 1:1 stoichiometry [(γ-CD)/C_70_] in the optimized geometry at the primary (top) and at the secondary rim (bottom), and facing it with a second γ-CD with the primary (at left) and the secondary rim (at right), as indicated in the two panels

We can follow the process of the complex γ-CD - C_70_ interacting with another γ-CD facing it with the primary and with the secondary rim as reported in Figure S2, during the initial 5 ns of MD run in vacuo in the animations:

[DYN_dimer_gCDs_C70_PP_5ns.avi](file:///C:\Users\ganazzoli.CHKNET\Documents\Ciclodestrine\Fullereni_inclusi\PaperC70\DYN_dimer_gCDs_C70_PP_5ns.avi),

[DYN_dimer_gCDs_C70_PS_5ns.avi](file:///C:\Users\ganazzoli.CHKNET\Documents\Ciclodestrine\Fullereni_inclusi\PaperC70\DYN_dimer_gCDs_C70_PS_5ns.avi),

[DYN_dimer_gCDs_C70_SP_5ns.avi](file:///C:\Users\ganazzoli.CHKNET\Documents\Ciclodestrine\Fullereni_inclusi\PaperC70\DYN_dimer_gCDs_C70_SP_5ns.avi),

[DYN_dimer_gCDs_C70_SS_5ns.avi](file:///C:\Users\ganazzoli.CHKNET\Documents\Ciclodestrine\Fullereni_inclusi\PaperC70\DYN_dimer_gCDs_C70_SS_5ns.avi), respectively.

**Complex Formation with a 2:1 Host-Guest Stoichiometry: [(γ-CD)_2_/C_70_] *in vacuo***


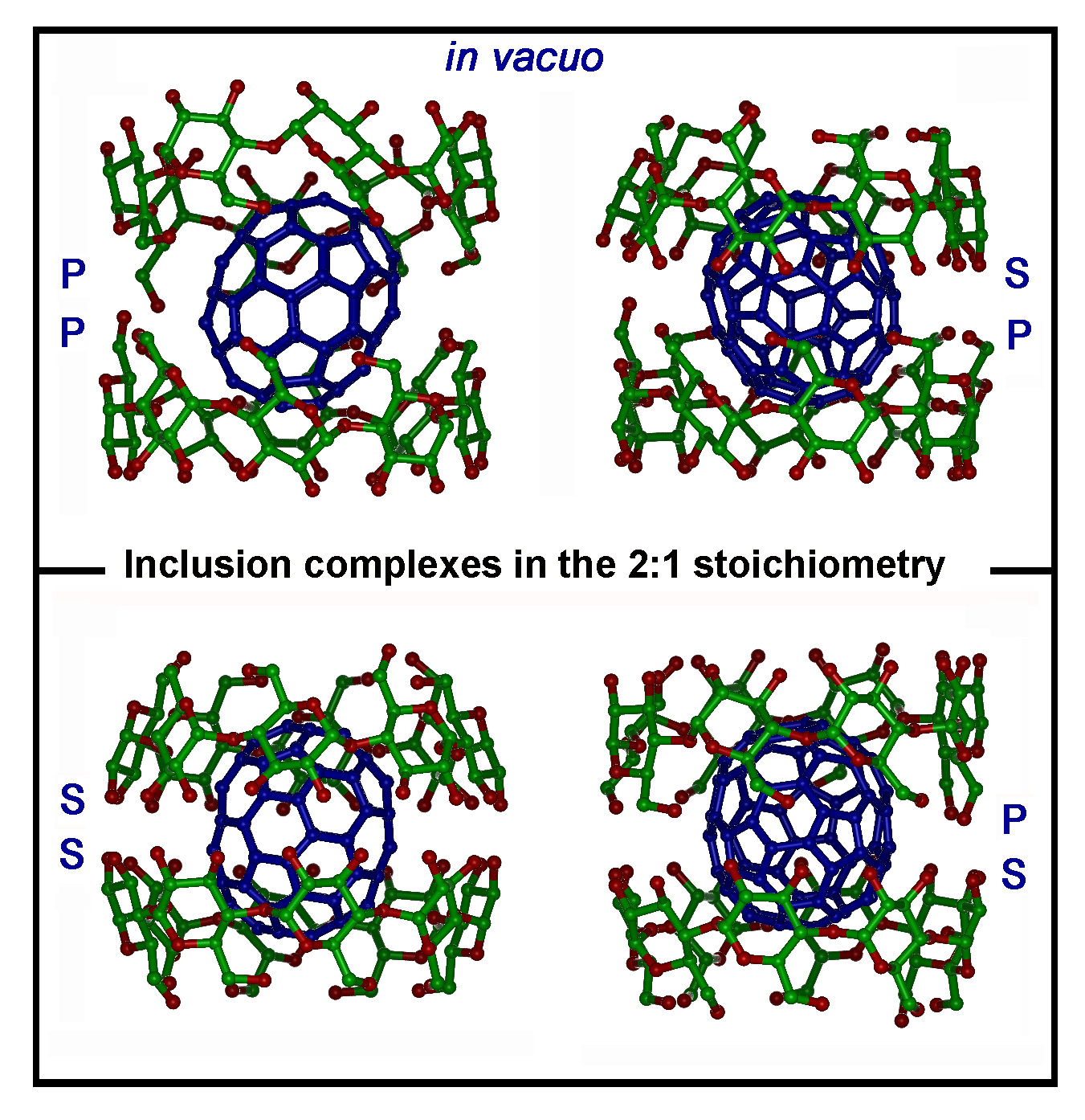


**Figure S3**. Side view of the most stable geometries of 2:1 Host-Guest Stoichiometry [(γ-CD)_2_/C_70_**]** found after the MD runs in vacuo at 300 K and optimization of numerous conformations (fifty conformations periodically saved during the MD run), starting from the initial complexes in the 1:1 stoichiometry (Figure S3), interacting with two different rims of the second γ-CD. Hydrogen atoms are omitted for clarity. See Figure S2 for the color codes.

Complex Formation with a 1:1 Host-Guest Stoichiometry: [(γ-CD)/C_70_] *in water*

We can follow the inclusion process of the C_70_ fullerene interacting with the primary and with the secondary rim of the γ-CD during the MD run in water lasting for 1 ns in the animation files

[dyn_in water_Gcd primary rim_C70.avi](file:///C:\Users\ganazzoli.CHKNET\Documents\Ciclodestrine\Fullereni_inclusi\PaperC70\dyn_in%20water_Gcd%20primary%20rim_C70.avi)

[dyn_in water_Gcd secondary rim_C70.avi](file:///C:\Users\ganazzoli.CHKNET\Documents\Ciclodestrine\Fullereni_inclusi\PaperC70\dyn_in%20water_Gcd%20secondary%20rim_C70.avi), respectively.

Complex Formation with a 2:1 Host-Guest Stoichiometry: [(γ-CD)_2_/C_70_] *in water*


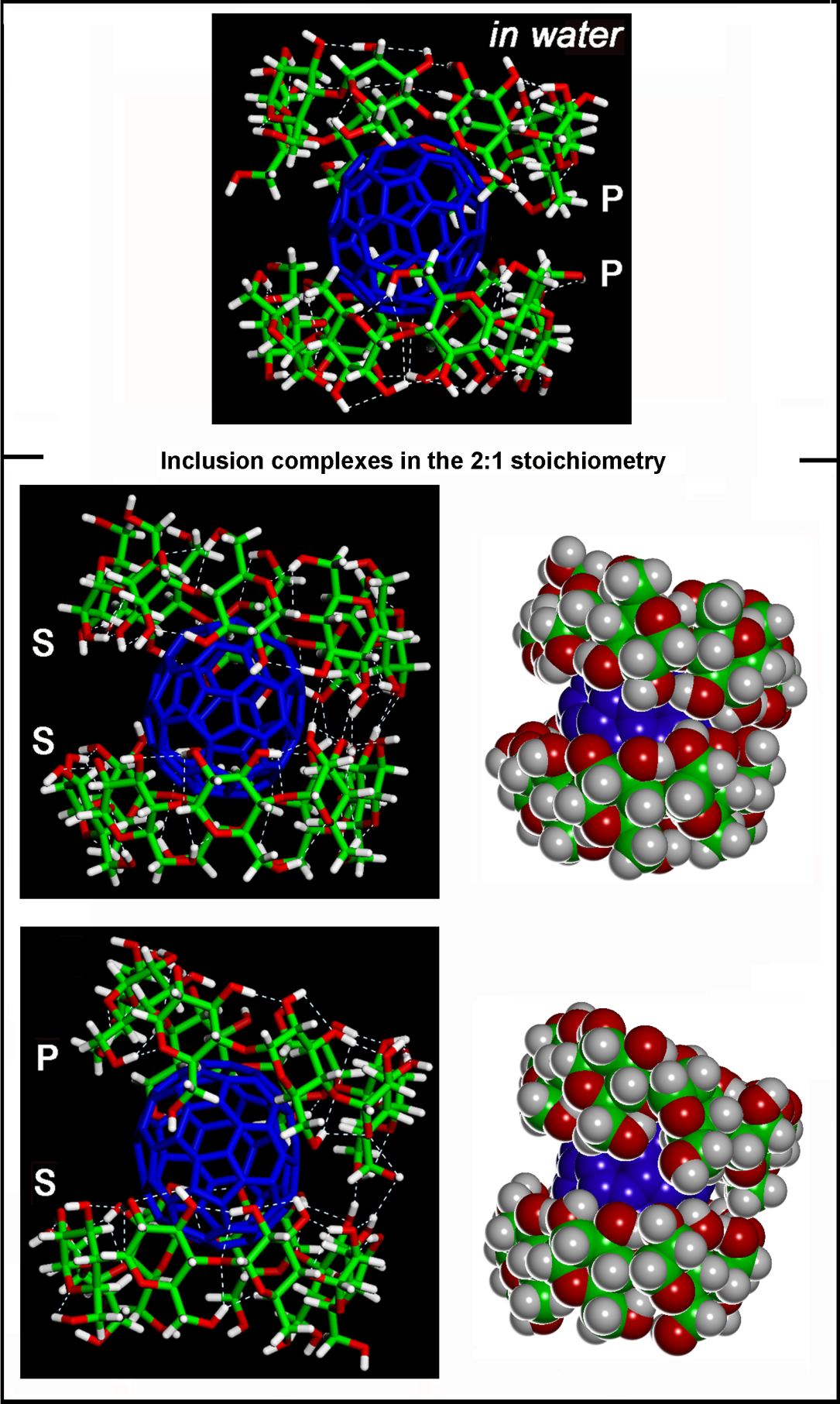


**Figure S4**. Stick side view of the final geometries obtained after MD runs lasting for 2 ns in water at 300 K and optimization of the conformation at equilibrium for the 2:1 complexes [(γ-CD)_2_/C_70_]. Hydrogen bonds are in white dotted lines. Water molecules and the simulation cells are omitted for clarity. The SS and SP complexes are reported using also the CPK representation. See Figure S2 for the color codes.

We can follow the formation of the 2:1 complex when the 1:1 complex, where C_70_ interacts with the secondary rim of the first γ-CD, is approached by the secondary or by the primary rim of the second γ-CD (respectively at left and at right of Figure 7 and in Figure S4), during the initial MD run in water in the animations files [DIM_SS_in water_500ps.avi](file:///C:\Users\ganazzoli.CHKNET\Documents\Ciclodestrine\Fullereni_inclusi\PaperC70\DIM_SS_in%20water_500ps.avi), [DIM_SP_in water_1ns.avi](file:///C:\Users\ganazzoli.CHKNET\Documents\Ciclodestrine\Fullereni_inclusi\PaperC70\DIM_SP_in%20water_1ns.avi), respectively. The same information about the most stable PP [(γ-CD)_2_/C_70_] complex in water as reported in Figure 6 is in the file [DIM_PP_in water_1ns.avi](file:///C:\Users\ganazzoli.CHKNET\Documents\Ciclodestrine\Fullereni_inclusi\PaperC70\DIM_PP_in%20water_1ns.avi).


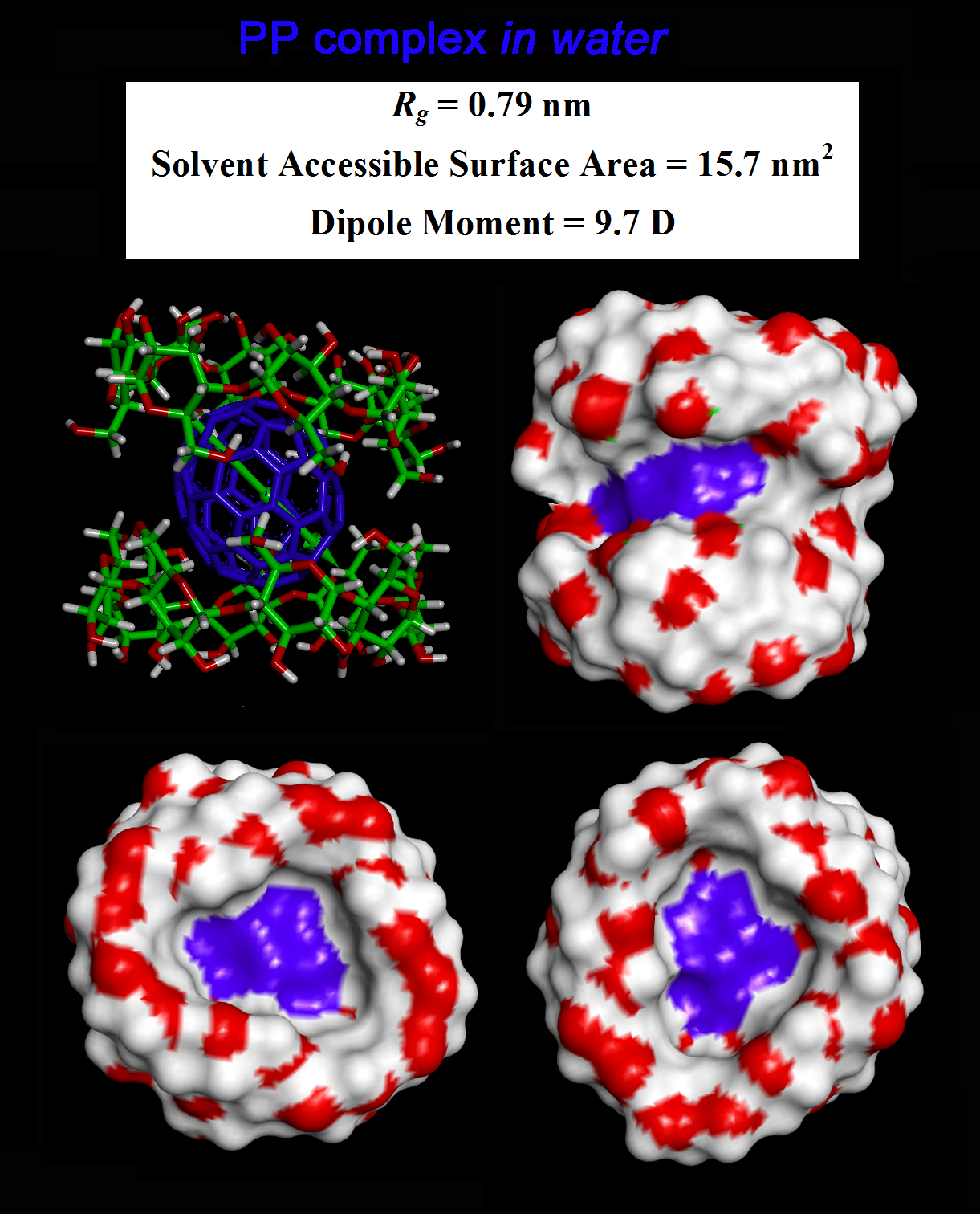


**Figure S5a**. Information about PP [(γ-CD)_2_/C_70_] complex in water. In the box, the values of the radius of gyration, R_g_, of the solvent accessible surface area and the dipole moment are reported. The figures show the dipole moment (on the top at left), the solvent accessible surface in the side view (on the top at right) and in the top views from the two secondary rims (below).


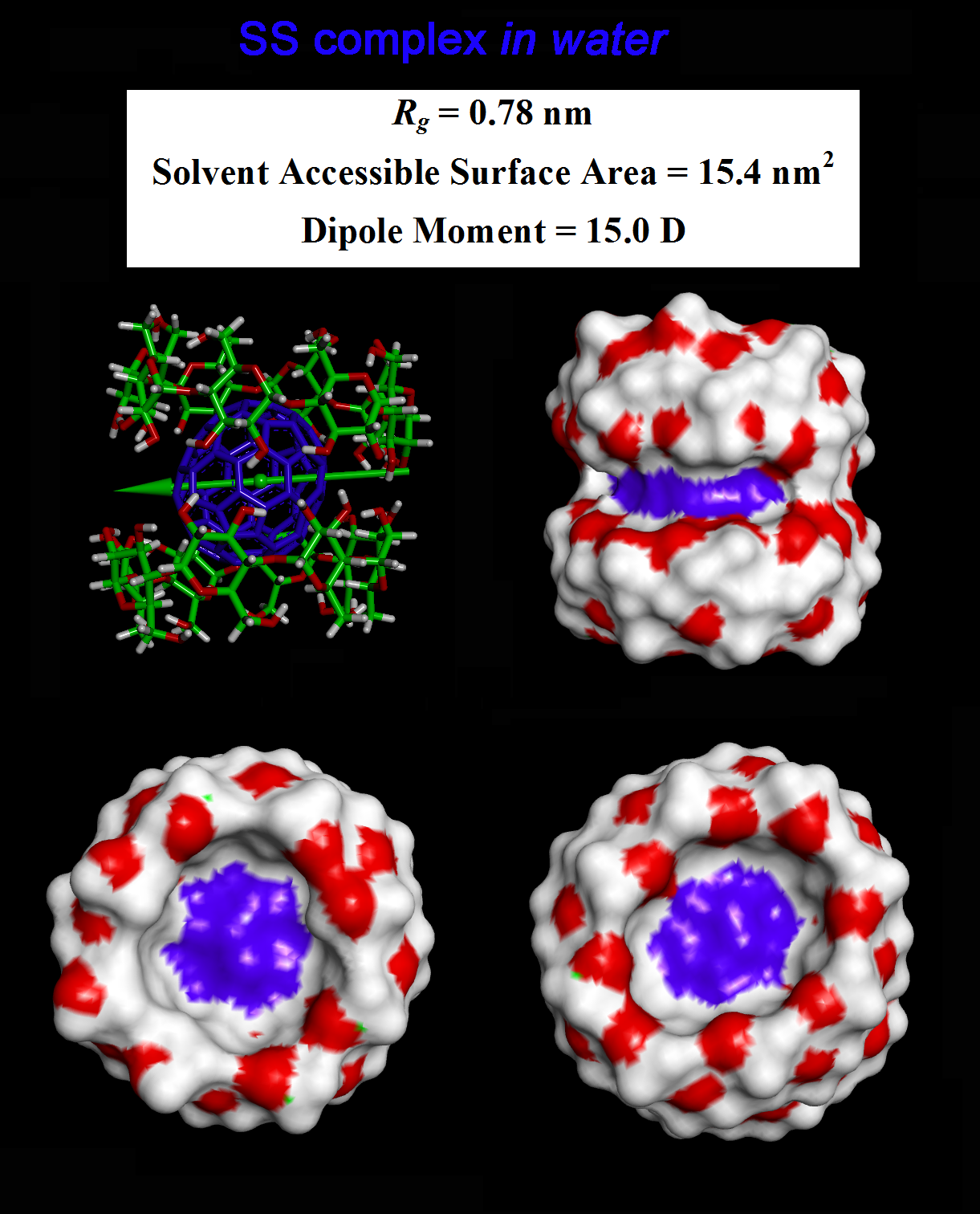


**Figure S5b**. Information about SS [(γ-CD)_2_/C_70_] complex in water. In the box, the values of the radius of gyration, R_g_, of the solvent accessible surface area and the dipole moment are reported. The figures show the dipole moment (on the top at left), the solvent accessible surface in the side view (on the top at right) and in the top views from the two secondary rims (below).


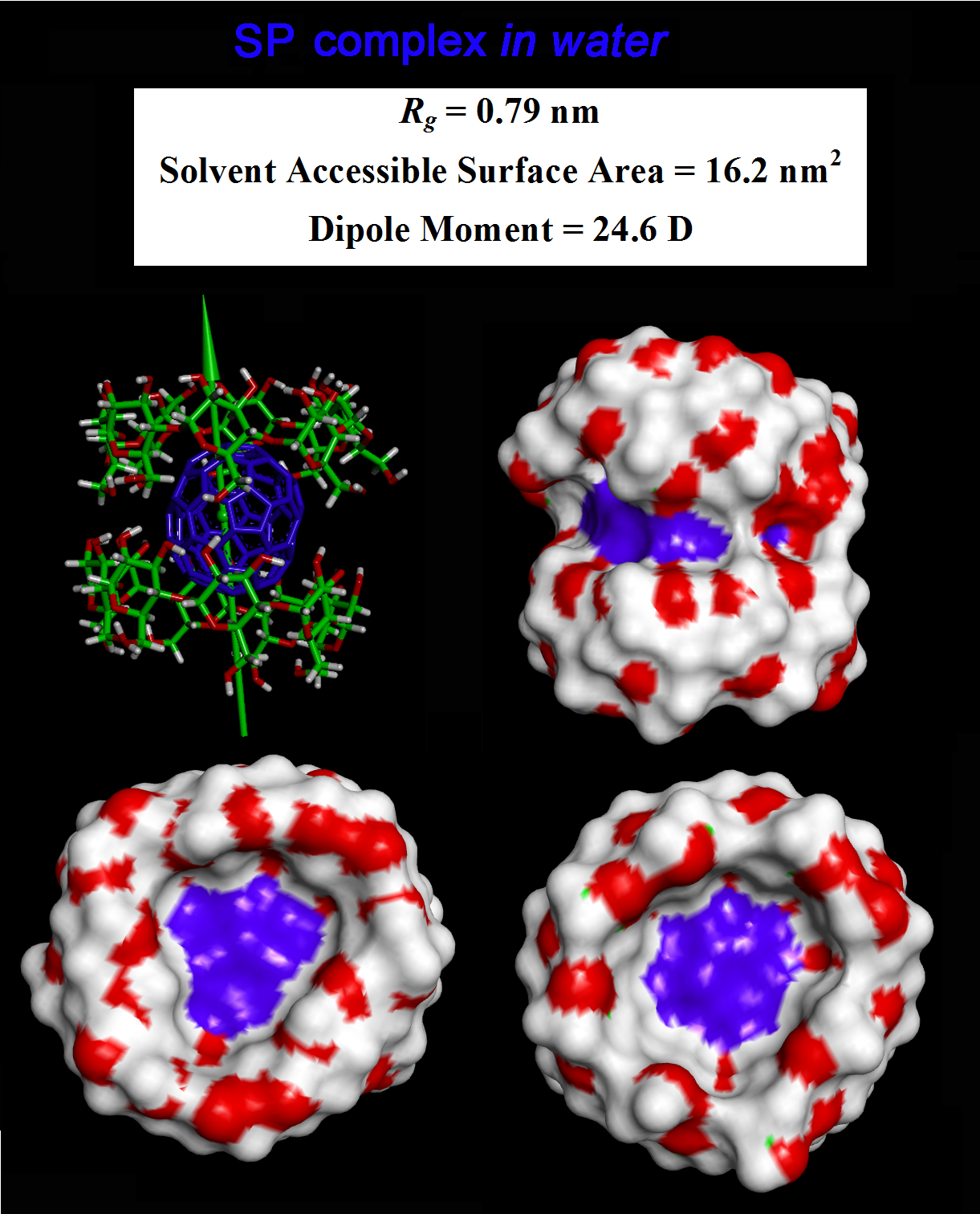


**Figure S5c**. Information about SP [(γ-CD)_2_/C_70_] complex in water. In the box, the values of the radius of gyration, R_g_, of the solvent accessible surface area and the dipole moment are reported. The figures show the dipole moment (on the top at left), the solvent accessible surface in the side view (on the top at right) and in the top views from the two secondary rims (below).
