# Supplementary figures and images for: A Molecular Dynamics Study of a Photodynamic Sensitizer for Cancer Cells: Inclusion Complexes of γ-Cyclodextrins with C70
Source: Int J Mol Sci. 2019 Sep 28;20(19):4831. doi: 10.3390/ijms20194831 (PMC6801912; doi:10.3390/ijms20194831)

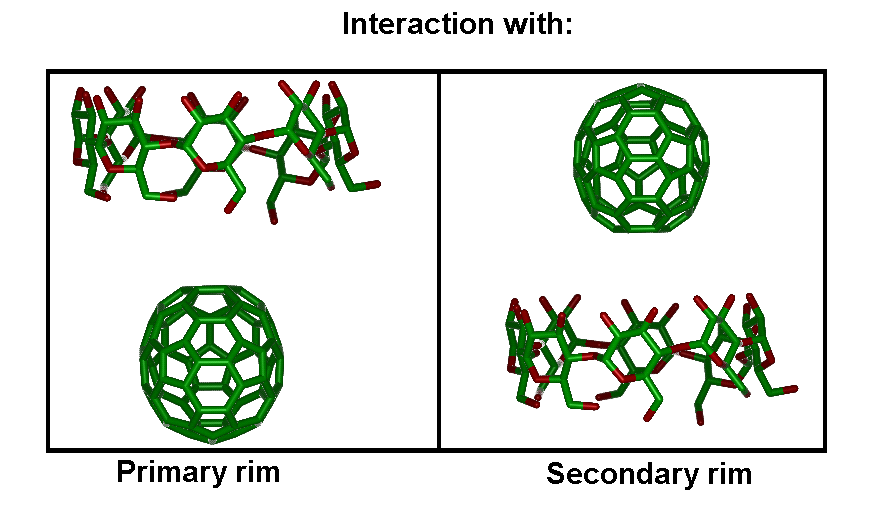

Supplement: Supplementary file 1 [file ijms-20-04831-s001.zip › Figure S1.tif]

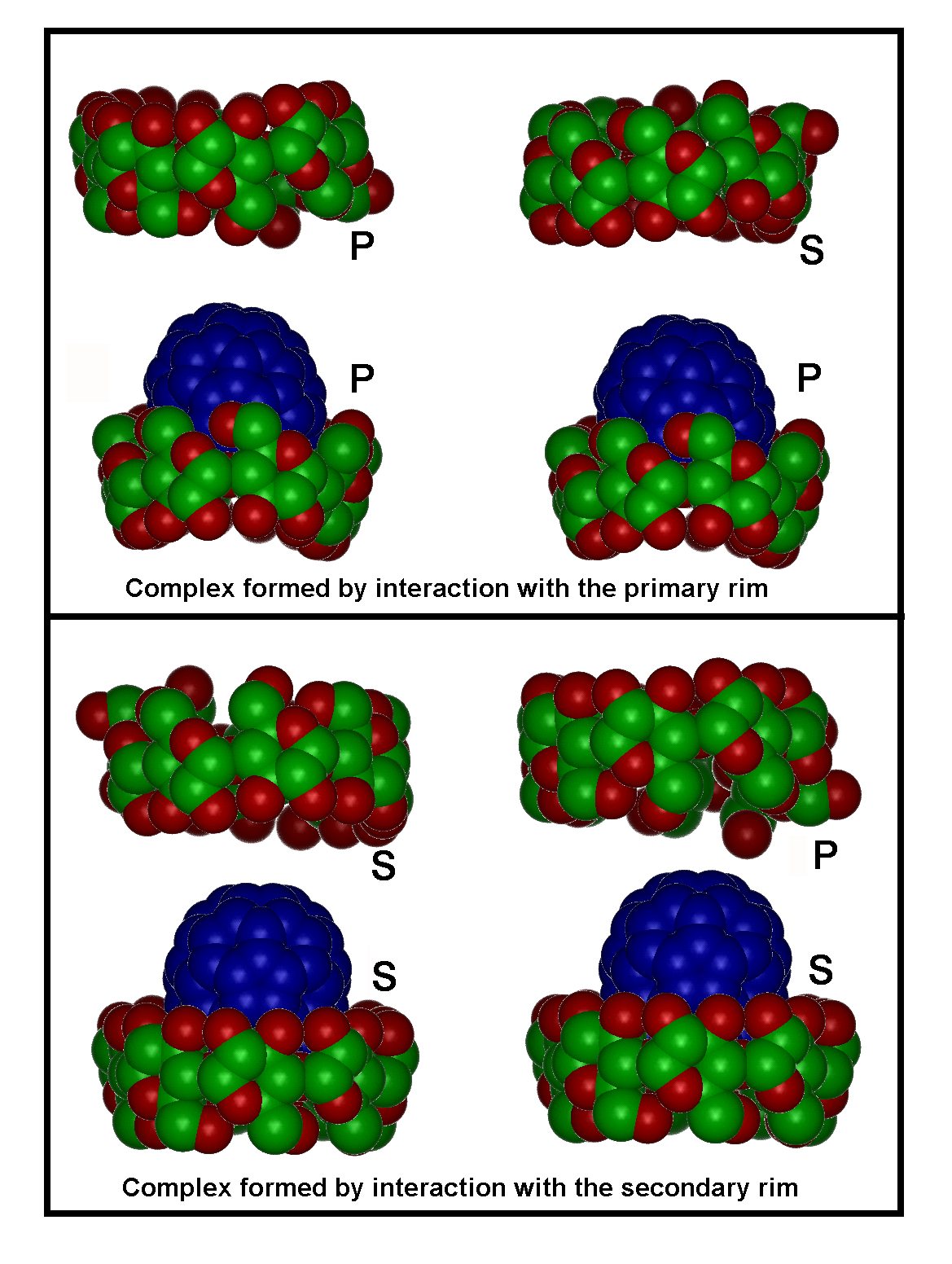

Supplement: Supplementary file 1 [file ijms-20-04831-s001.zip › Figure S2.tif]

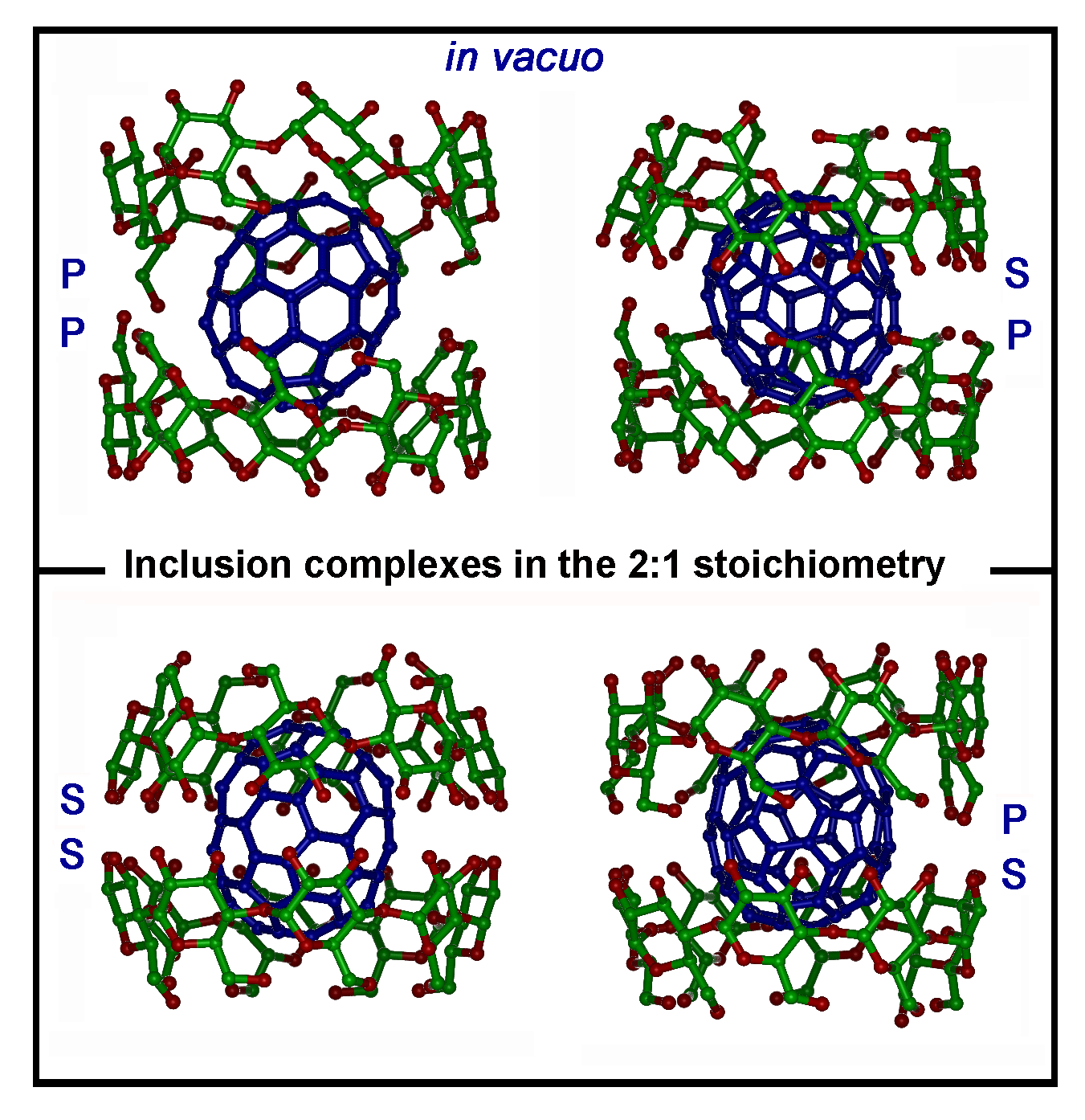

Supplement: Supplementary file 1 [file ijms-20-04831-s001.zip › Figure S3.tif]

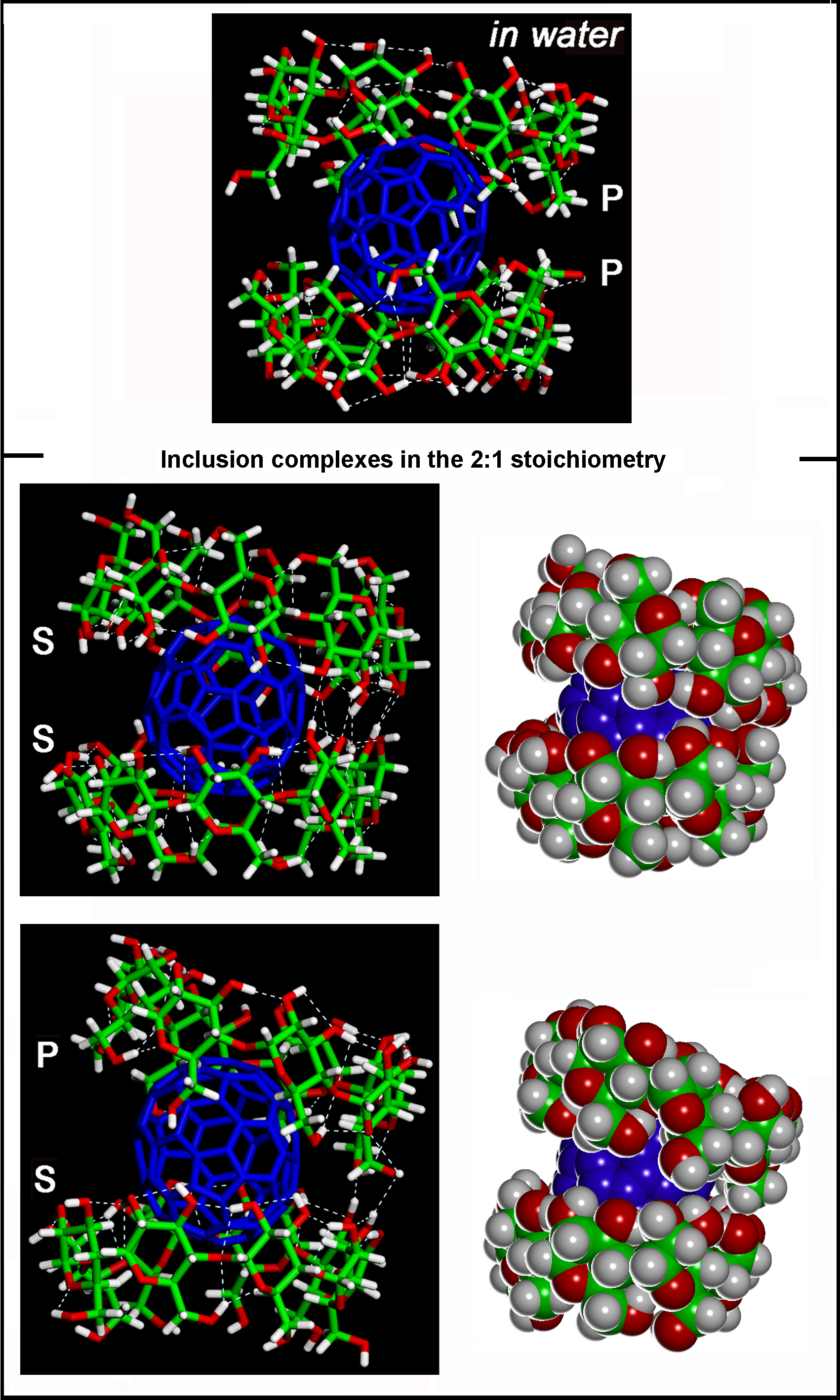

Supplement: Supplementary file 1 [file ijms-20-04831-s001.zip › Figure S4.tif]

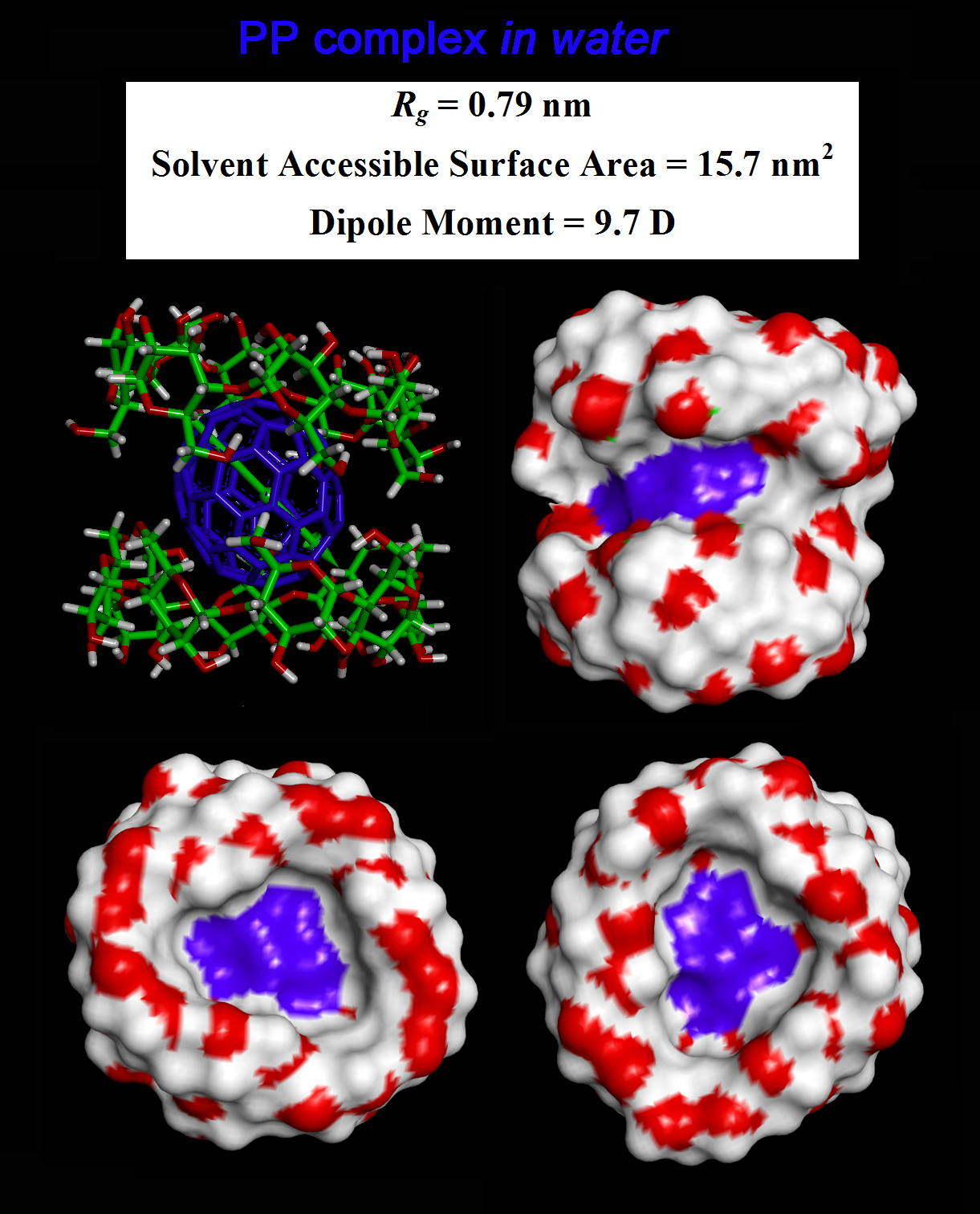

Supplement: Supplementary file 1 [file ijms-20-04831-s001.zip › Figure S5a.tif]

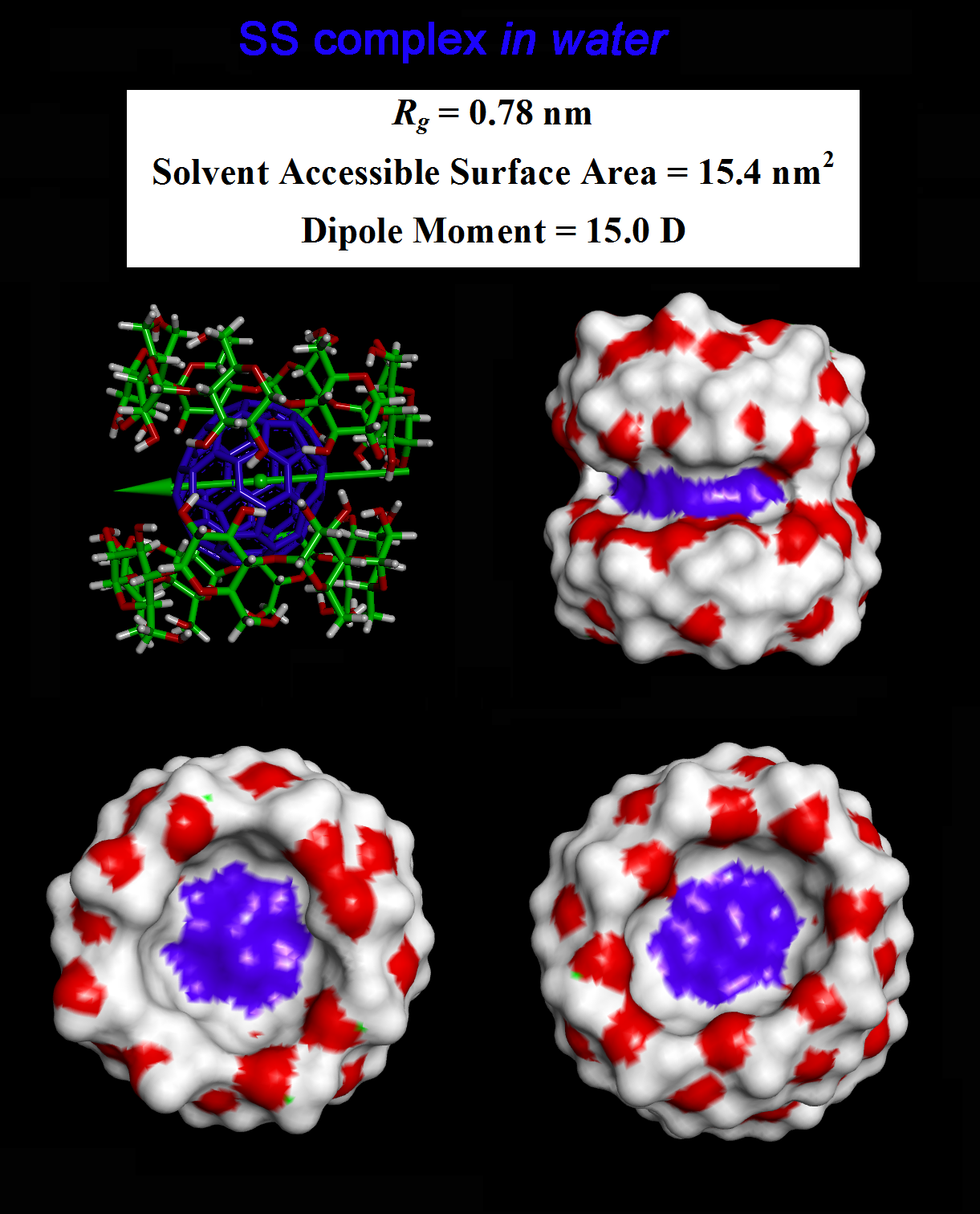

Supplement: Supplementary file 1 [file ijms-20-04831-s001.zip › Figure S5b.tif]

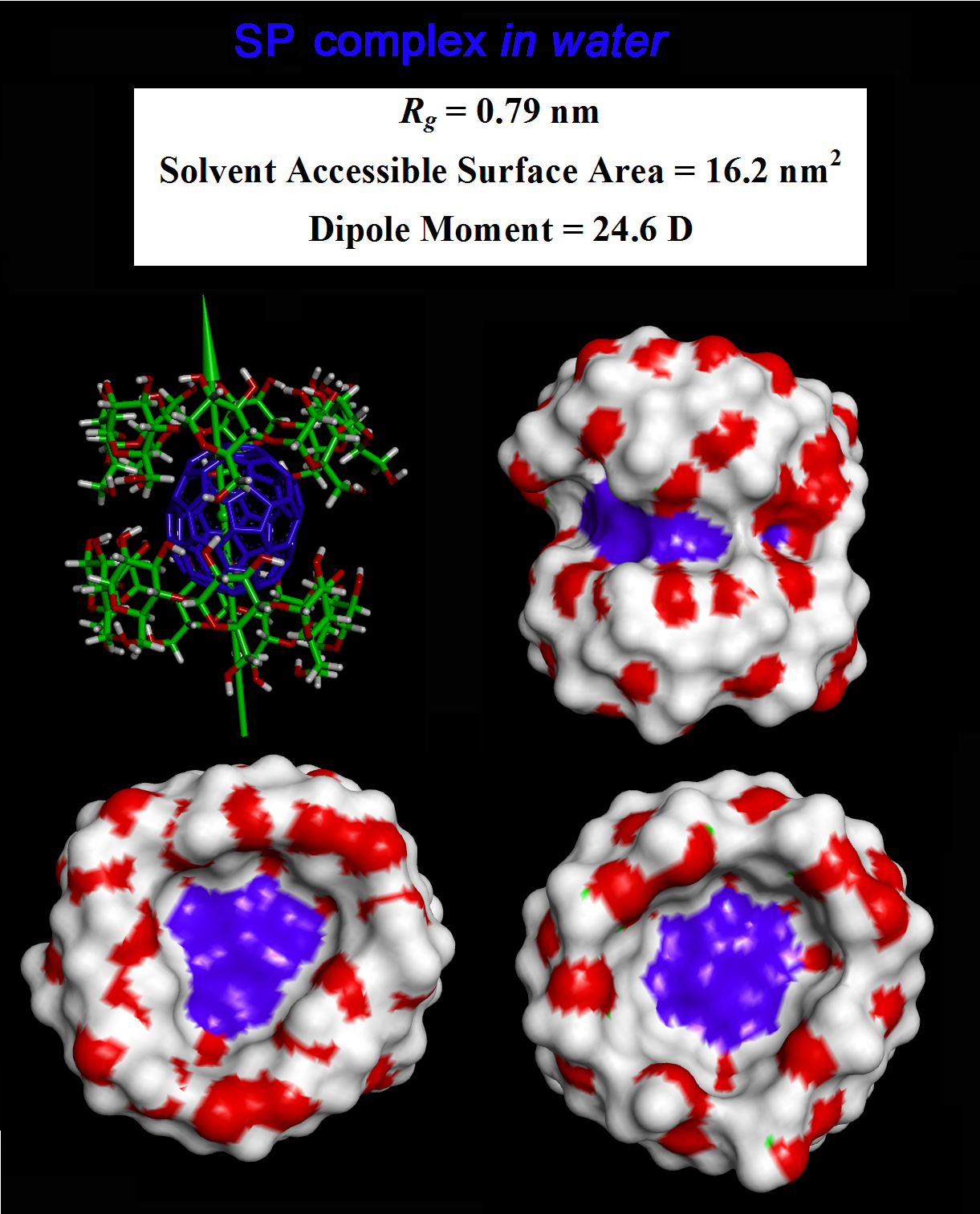

Supplement: Supplementary file 1 [file ijms-20-04831-s001.zip › Figure S5c.tif]
